# *Cryptocarya kaengkrachanensis*, a new species of Lauraceae from Kaeng Krachan National Park, southwest Thailand

**DOI:** 10.3897/phytokeys.140.34574

**Published:** 2020-03-04

**Authors:** Meng Zhang, Tetsukazu Yahara, Shuichiro Tagane, Sukid Rueangruea, Somran Suddee, Etsuko Moritsuka, Yoshihisa Suyama

**Affiliations:** 1 Department of Biology, Kyushu University, 744 Motooka, Fukuoka, 819-0395, Japan Kyushu University Fukuoka Japan; 2 The Kagoshima University Museum, Kagoshima University, 1-21-30 Korimoto, Kagoshima, 890-0065, Japan Kagoshima University Kagoshima Japan; 3 Forest Herbarium, Department of National Parks, Wildlife and Plant Conservation, Chatuchak, Bangkok, 10900, Thailand Department of National Parks Bangkok Thailand; 4 Kawatabi Field Science Center, Graduate School of Agricultural Science, Tohoku University, 232-3 Yomogida, Naruko-onsen, Osaki, Miyagi 989-6711, Japan Tohoku University Sendai Japan

**Keywords:** *
Cryptocarya
*, flora, Laurales, Lauraceae, new species, taxonomy, Thailand

## Abstract

A new species of Lauraceae, *Cryptocarya kaengkrachanensis* M.Z.Zhang, Yahara & Tagane, from Kaeng Krachan National Park, Phetchaburi Province, southwestern Thailand, is described and illustrated. This species is morphologically most similar to *C. amygdalina* in that its leaves are pinnately veined, leathery, and apparently glabrous (but microscopically hairy) abaxially, twigs are yellowish brown hairy, and fruits are 1.36 to 1.85 times longer than width. However, *C. kaengkrachanensis* is distinguished from *C. amygdalina* in having the leaves of ovate and elliptic (vs. oblong-lanceolate) with leaf aspect ratio (length:width) from 1.38 to 2.28 (vs. 2.46–3.43), and ovoid fruits (vs. ellipsoid) with stalk distinctly swollen (vs. not or only slightly swollen). In addition, phylogenetic trees constructed based on internal transcribed spacer sequences (ITS) and genome-wide SNPs using MIG-seq showed that *C. kaengkrachanensis* is not sister to *C. amygdalina* and is distinct from all the other *Cryptocarya* species hitherto recognized in Thailand. Analysis including other species demonstrates that *C. floribunda* should be a synonym of *C. amygdalina*, but we recognize *C. scortechinii* as a distinct species.

## Introduction

Lauraceae, a plant family widely distributed across the world, contain an estimated 2500–3500 species in about 50 genera, and its highest species richness is found in the tropical forests of Southeast Asia and the Americas (Rohwer 1993, Li et al. 2008, Rohwer et al. 2014, Yahara et al. 2016). In Southeast Asia, trees of Lauraceae occur widely from lowlands to high elevations (van der Werff 2001, Ngernsaengsaruay et al. 2011, Wuu-Kuang 2011, de Kok 2015, 2016a, 2016b, Yahara et al. 2016), and are often among the most dominant components of the canopy in montane forests (Ohsawa 1991, Tagawa 1995, Sri-Ngernyuang et al. 2003). Reflecting their species diversity and dominance, many taxonomic studies have been published for Lauraceae of Southeast Asia, including a classic monograph by Liou (1934), a series of publications by Kostermans (1968, 1969, 1970, 1974, 1988), floristic treatments of Lecomte (1914), Kochummen (1989), and Hô (1999), and more recent publications of new species by various authors (Nishida 2008, Liu et al. 2013, Tagane et al. 2015, de Kok 2016a, 2016b, Yahara et al. 2016, Mitsuyuki et al. 2018). In spite of all these studies, the species-level taxonomy of Lauraceae is still in need of critical scrutiny in many parts of Southeast Asia (Yahara et al. 2016).

Among Southeast Asian genera of Lauraceae, the genus *Cryptocarya* is particularly well studied. de Kok (2015) revised the taxonomy of *Cryptocarya* of Indochina and Thailand and enumerated 16 species, among which six species are endemic to Indochina and Thailand. Subsequently, de Kok (2016a) revised *Cryptocarya* of Peninsular Malaysia and recognized 17 species, among which three species are endemic to Peninsular Malaysia. However, during our field surveys in Cambodia, Laos, Vietnam, Thailand, Myanmar, Malaysia and Indonesia, we collected specimens of *Cryptocarya* that are difficult to identify using the classifications of de Kok (2015, 2016a). By combining molecular phylogenetic evidence, comparative morphological studies, and field observations on sympatric occurrences of different entities, we concluded that some of these represent new species. In this paper, we document three species of *Cryptocarya* as occurring in Kaeng Krachan National Park, Phetchaburi Province, southwest Thailand, namely *C. amygdalina* Nees, *C. pustulata* Kosterm. and a new species, described below.

According to a taxonomic treatment of de Kok (2015, 2016a), two entities we collected in Kaeng Krachan National Park, excluding *C. pustulata*, keyed out as *C. amygdalina*. This name is arrived at because (1) the leaves are elliptic, pinnately veined and leathery; (2) the mature lower leaf surface is apparently glabrous except on veins (but microscopically hairy), (3) young twigs are covered with yellowish brown hairs, and (4) mature fruits are ellipsoid or ovoid (not globose) and smooth (not ridged). *Cryptocarya amygdalinas. str.* is a species described from India, but de Kok (2015, 2016a) proposed a broader concept of *C. amygdalina* by including *C. floribunda* Nees described from Bangladesh and *C. scortechinii* Gamble described from Peninsular Malaysia. However, the discovery of what turned out to be two co-occurring species in Kaeng Krachan National Park identified as “*C. amygdalina*” following the classification system of de Kok (2015, 2016a) lead us to reassess his broader concept of “*C. amygdalina*”. Here, we show that the two species from Kaeng Krachan National Park identified as “*C. amygdalina*” are not sister to each other in a phylogenetic tree constructed by ITS sequences and genome-wide SNPs of MIG-seq (Suyama and Matsuki 2015). By combining the molecular evidence with morphological and field observations, we revise the broader concept of “*C. amygdalina*” of de Kok (2015, 2016a) by recognizing three species, *C. amygdalina* s. str., *C. scortechinii*, and a taxon from Kaeng Krachen National Park.

## Materials and methods

### Field observations

**Figure 1.**
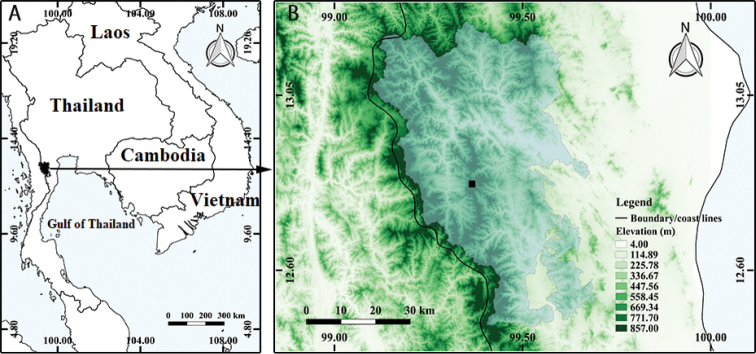
Study area **A** location of Kaeng Krachan National Park **B** topographical map of Kaeng Krachan National Park; solid square indicates the type locality (elevation, 960 m) of *Cryptocarya kaengkrachanensis* M.Z. Zhang, Yahara & Tagane.

In Kaeng Krachan National Park (Fig. 1), we established five 100 m × 5 m plots at elevations of 360 m (12°48'11.4"N, 99°26'31.6"E, surveyed on 5 Oct. 2012), 540 m (12°48'18.48"N, 99°25'07.12"E, surveyed on 27 May 2014), 680 m (12°48'25.6"N, 99°24'24.0"E, surveyed on 25 Oct. 2013), 850 m (12°49'03.5"N, 99°22'53.5"E, surveyed on 28 Oct. 2013), and 960 m (12°49'19.7"N, 99°21'57.7"E, surveyed on 21 Oct. 2013). All vascular plants were recorded in each plot. For trees 4 m or taller, we recorded girth and height of trunks. For trees lower than 4 m and herbs, we recorded presence/absence in each of ten 10 m × 5 m sections. For all the species distinguished in the field, we collected voucher specimens and sampled some pieces of leaves for DNA isolation (vouchers were deposited at BKF and FU). Each sample collected for DNA isolation was dried with silica gel in a zipper storage bag. In addition to plants recorded in the five plots, we also collected additional specimens with flowers or fruits from outside the plots.

### Morphological observation

We scanned dried herbarium specimens and specimen images were measured for leaf length, leaf width, aspect ratio and circularity using ImageJ (Schneider et al. 2012); aspect ratio and circularity are defined as leaf length/leaf width and 4π × (area/perimeter squared), respectively. To identify species, we examined images of type specimens using JSTOR Global Plants (https://plants.jstor.org/). We also examined specimens kept at the herbaria BKF, BO, FOF, FU, KAG, KYO, RUPP, SAR, SNP and VNM, and reviewed taxonomic literature of *Cryptocarya* in Southeast Asia (Lecomte 1914, Liou 1934, Kostermans 1988, Kochummen 1989, Hô 1999, Dy Phon 2000, Newman et al. 2007, Li et al. 2008, de Kok 2015, 2016a, Liu et al. 2017).

### DNA barcoding

For DNA extraction, we milled the dried leaf material into fine powder by QIAGEN TissueLyser and the powder was washed three times with 1 ml buffer solution (including 0.1 M HEPES, pH 8.0; 2% Mercaptoethanol; 1% PVP; 0.05 M Ascorbic acid) (Toyama et al. 2015). DNA was then isolated from the washed powder by using the CTAB method (Doyle and Doyle 1987) with a slight modification (Toyama et al. 2015).

We determined partial sequences of the internal transcribed spacer (ITS region) of ribosomal DNA using the following primer sets of Rohwer et al. (2009): ITS18-F (5’-GTCCACTGAACCTTATCATTTAGAGG-3’) and ITS26-R (5’-GCCGTTACTAAGGGAATCCTTGTTAG-3’) and Tks Gﬂex DNA Polymerase (Takara Bio, Kusatsu, Japan) (Binh et al. 2018). The PCR reaction was carried out following the published protocols of Kress et al. (2009) with some modification by setting PCR cycling conditions as [95 °C 4 min (94 °C 30 sec, 55 °C 1 min, 72 °C 1 min) 25 cycles, 72 °C 10 min]. The PCR products were purified by a diluted mixture of ExoSap-IT (GE Healthcare, Little Chalfont, UK). Using the purified PCR products, forward and reverse sequencing was carried out separately by adding BigDye terminator sequencing mixture (BigDye Terminator v3.1; Applied Biosystems) and setting cycling conditions as [96 °C 1 min (96 °C 30 sec, 50 °C 30 sec, 60 °C 4 min) 25 cycles]. The BigDye reaction products were finally read with ABI 3730 automated sequencer (Applied Biosystems, Foster City, CA, USA).

We determined ITS sequences for 29 samples of *Cryptocarya* (Table 1) including two samples (numbered as T1883 and T2069) of the Kaeng Krachan taxon and 27 samples of eleven known species. All of these sequences are deposited into the DDBJ database with accession numbers in Table 1. In addition, we downloaded one sequence of a *Beilschmiedia* sp. collected in Vietnam from the NCBI database (http://www.ncbi.nlm.nih.gov) for outgroup comparison (Table 1).

**Table 1.** Sample lists for genotyping.

**Country**	**Area**	**Sample ID**	**DDBJ Acc. No.**	**Specimen**	**Species or variety**
Cambodia	Cardamon	625	LC479107	625 (FU)	*C. concinna*
	Cardamon	657	LC479108	657 (FU)	*C. concinna*
	Bokor	1839	LC479106	1839 (FU)	*C. concinna*
	Bokor	6217	LC479111	6217 (FU)	*C. concinna*
Laos	Nam Kading	L21	LC477686	L21 (FU)	*C. sublanuginosa*
	Nam Kading	L26	LC477687	L26 (FU)	*C. sublanuginosa*
	Nam Kading	L49	LC477688	L49 (FU)	*C. sublanuginosa*
Myanmar	Tanintharyi	MY479	LC477685	MY479 (FU)	*C. amygdalina*
Thailand	Doi Inthanon	T5	LC479104	T5 (FU)	*C. kurzii*
	Doi Inthanon	T16	LC479117	T16 (FU)	*C. densiflora*
	Doi Inthanon	T1373	LC479118	T1373 (FU)	*C. densiflora*
	Khao Soi Dao	T1545	LC479098	T1545 (FU)	*C. pustulata*
	Kaeng Krachan	T1883	LC405942	T1883 (FU)	*C. kaengkrachanensis*
	Kaeng Krachan	T2069	LC405941	T2069 (FU)	*C. kaengkrachanensis*
	Kaeng Krachan	T2195	LC479099	T2195 (FU)	*C. pustulata*
	Khao Soi Dao	T2838	LC479097	T2838 (FU)	*C. chanthaburiensis*
	Kaeng Krachan	T2971	LC479100	T2971 (FU)	*C. pustulata*
	Kaeng Krachan	T3090	LC477684	T3090 (FU)	*C. amygdalina*
	Phu Kradueng	T3589	LC479102	T3589 (FU)	*C. pallens*
	Khao Luang	T3902	LC479101	T3902 (FU)	*C. albiramea*
	Khao Luang	T3944	LC479116	T3944 (FU)	*C. densiflora*
	Phu Kradueng	T4471	LC479105	T4471 (FU)	*C. kurzii*
	Phu Kradueng	T4507	LC479103	T4507 (FU)	*C. kurzii*
	Khao Luang	T4796	LC479115	T4796 (FU)	*C. scortechinii*
Vietnam	Bach Ma	V2462	LC479112	V2462 (FU)	*C. concinna*
	Bach Ma	V3287	LC479109	V3287 (FU)	*C. concinna*
	Vu Quang	V3518	LC479114	V3518 (FU)	*C. concinna*
	Vu Quang	V3566	LC479113	V3566 (FU)	*C. concinna*
	Vu Quang	V5615	LC479110	V5615 (FU)	*C. concinna*
Vietnam	–	HG315547.1	HG315547.1	–	*Beilschmiedia* sp.

### Next generation DNA sequencing – MIG-seq

We amplified thousands of short sequences by using the primers of “multiplexed ISSR (inter simple sequence repeats) genotyping by sequencing” (MIG-seq, Suyama and Matsuki 2015) for 24 samples of *Cryptocarya*, following the protocol of Suyama and Matsuki (2015). Two steps of PCR were performed; for the 1^st^ PCR step, we amplified ISSR regions from genomic DNA with MIG-seq tailed ISSR primer set-1 and diluted 50 times for each 1^st^ PCR product with deionized water (Suyama and Matsuki 2015, Binh et al. 2018). The 2^nd^ PCR step was conducted with common and indexed primers. The 2^nd^ PCR products were then pooled in equimolar concentrations as a single mixture library. Fragments of size range 350–800 bp were isolated from the purified mixture of 2^nd^ PCR products by a Pippin Prep DNA size selection system (Sage Science, Beverly, MA, USA) The concentration was measured by quantitative PCR (Library Quantification Kit; Clontech Laboratories, Mountain View, CA, USA) and then sequenced by Illumina MiSeq Sequencer (Illumina, San Diego, CA, USA) with a MiSeq Reagent Kit v3 (150 cycle, Illumina) (Suyama and Matsuki 2015, Binh et al. 2018).

### Phylogenetic tree reconstruction

For DNA barcoding analysis, MEGA X (Kumar et al. 2018, http://www.megasoftware.net/) was used to assemble the ITS sequences of 30 samples including 29 of *Cryptocarya* spp. and an additional sample of *Beilschmiedia* sp.; MAFFT ver. 7 (http://mafft.cbrc.jp) was used to align ITS sequences. We reconstructed a phylogenetic tree by the maximum likelihood method with Tamura 3-parameter model using MEGA X with a bootstrap test of 1500 replicates. In addition, we drew a TCS haplotype network (Clement et al. 2000) among 29 samples of *Cryptocarya* using POPART ver.1.7 (Leigh and Bryant 2015).

For MIG-seq, we pretreated the raw data of *Cryptocarya* samples following the quality control protocol of Suyama and Matsuki (2015) and Binh et al. (2018). We then assembled homologous sequences (designated as loci below) with the *de novo* map pipelines (ustacks, cstacks, sstacks) using Stacks ver. 1.48 (Catchen et al. 2011). First, we assembled loci by ustacks with the following settings: *m* = 3, *M* = 3, *N* = 2, and maximum gaps = 2 (where “*m*” is the minimum depth of coverage, “*M*” is maximum distance allowed between stacks, and “*N*” is the maximum distance allowed to align secondary reads to primary stacks). We then used cstacks to build a catalogue of consensus loci by assembling loci from ustacks, by setting the parameter of “number of allowed mismatches between sample (n)” as 2. Second, by using the sstacks, we associated all stacks created by ustacks with the catalog produced by cstacks. Third, we got an output vcf file containing genotypes of individuals at each locus. Subsequently, we used the vcf2phylip program (Ortiz 2019) to convert the vcf file to a phylip type file. Finally, we constructed a maximum likelihood tree with RAxML ver. 8.2 (Stamatakis 2014) and examined its reliability by bootstrapping using 1500 replicates.

## Results

### Field observation

In the plot at an elevation of 360 m, we recorded three sterile trees of *C. pustulata* and collected a specimen (voucher specimen number T0524) from one of these trees. In the plot at 540 m, we found no trees of *Cryptocarya*. However, a sterile specimen of *C. pustulata* was collected along the roadside at 550 m (T2971). In the plot at 680 m, we recorded two sterile trees of *C. pustulata* for which we recorded girth × height as 110.7 cm × 25 m and 11.3 cm × 5.5 m, respectively. In addition, we collected a sterile specimen (T2195) from a tree lower than 4 m. Along the roadside at 709 m, we collected a fruiting specimen of *C. amygdalina* (T3090) on 30 May 2014. In the plot at 850 m, we recorded two sterile trees of *Cryptocarya*, both of which were lower than 4 m. However, we could not identify these trees and vouchers were not collected. In the plot at 960 m (Fig. 1), we recorded girth × height for two trees of the Kaeng Krachan taxon as 24.3 cm × 5 m and 15.7 × 4.5 m, respectively. In addition, in the vicinity of the plot, a fruiting specimen (T2069) was collected from a tree 12 m tall on 23 Oct. 2013. Young trees of the Kaeng Krachan taxon lower than 4 m were found in all ten sections of 10 m × 5 m in the 100 m × 5 m plot at the elevation of 960 m.

### Morphological observation

**Figure 2.**
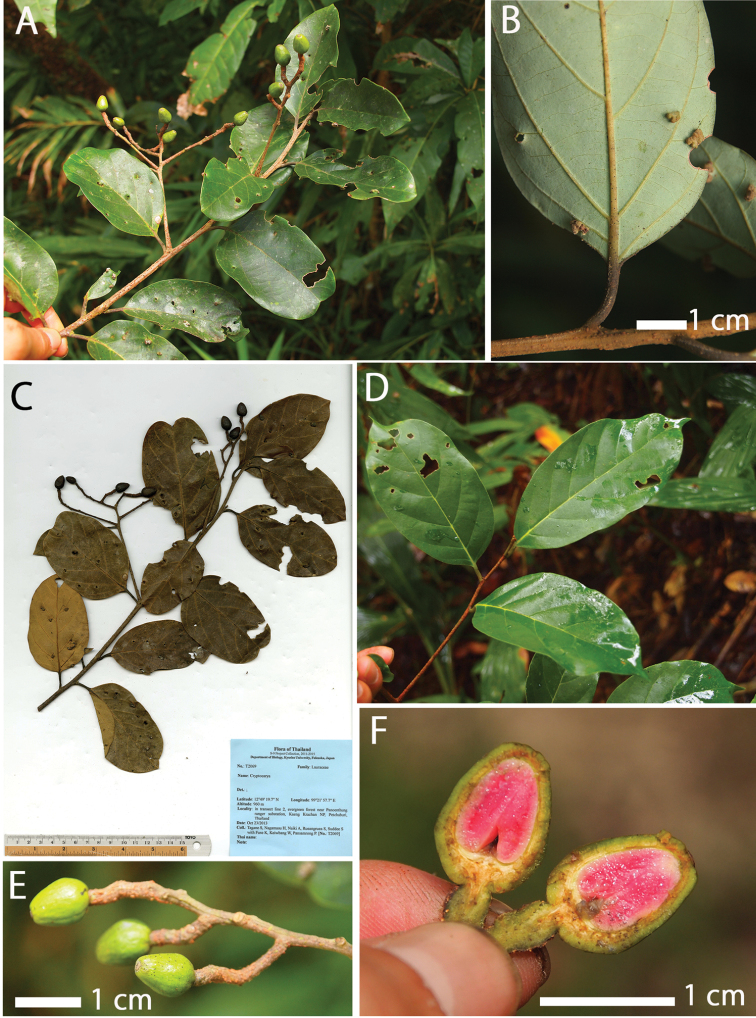
*Cryptocarya kaengkrachanensis* M.Z. Zhang, Yahara & Tagane **A** branch with immature fruit **B** lower leaf surface **C** holotype: *Tagane et al*. *T2069* (KYO) **D** young branchlet **E** part of an infructescence with immature fruits **F** longitudinal sections of an immature fruit.

**Figure 3.**
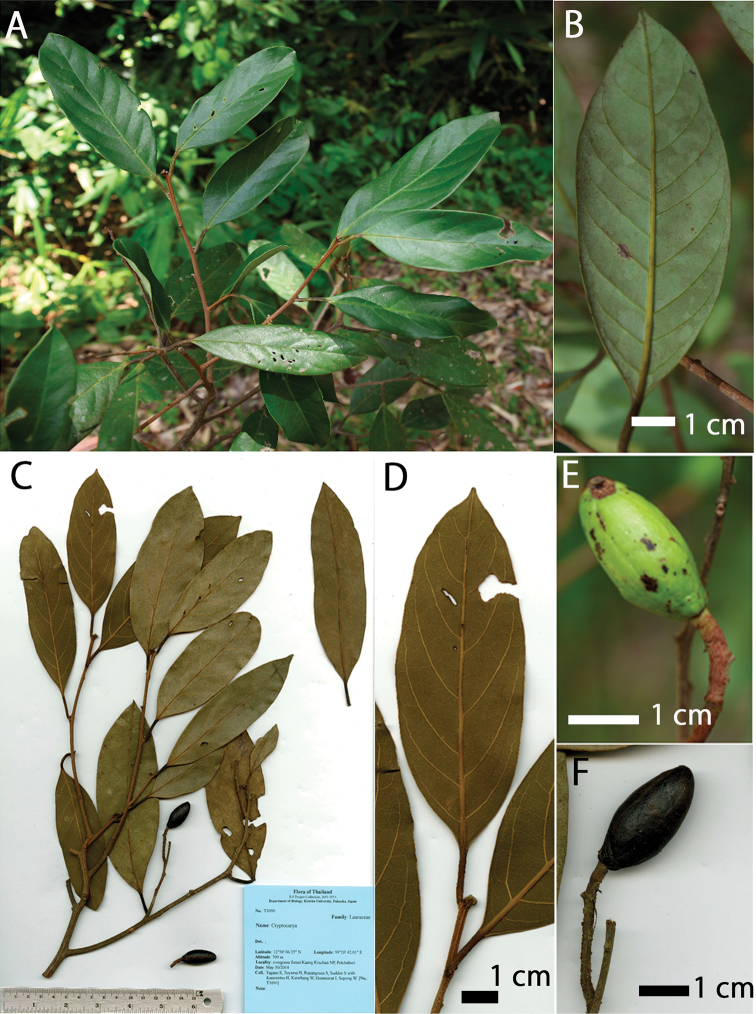
*Cryptocarya amygdalina***A** leafy branchlet **B** lower leaf surface **C** specimen *Tagane et al*. *T3090* (KYO) **D** lower leaf surface (dry) **E** fresh immature fruit **F** dried fruit.

**Figure 4.**
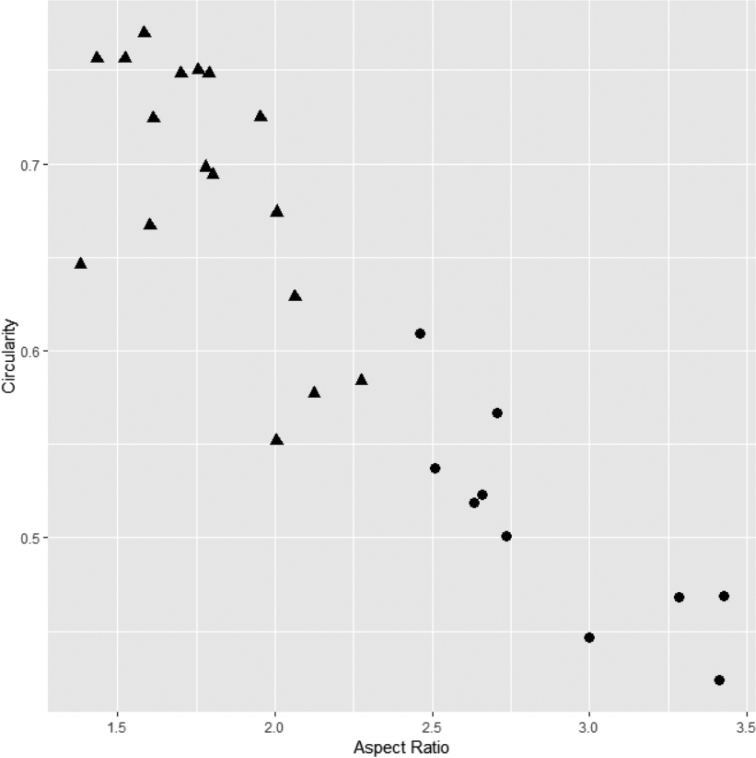
Scatter plot of leaf aspect ratio (horizontal axis) and circularity (vertical axis). Solid circles: *Cryptocarya amygdalina*, solid triangles: *C. kaengkrachanensis*.

In fruiting specimens, the Kaeng Krachan taxon (T2069; Fig. 2) has relatively shorter and broader leaves than *C. amygdalina* (T3090; Fig. 3), but the ranges are largely overlapping: the range (and average±SD) of leaf length (cm) is 2.6–10.3 (7.2±2.5) in the Kaeng Krachan taxon (n=17) vs. 9.4–14.1 (11.6±1.4) in *C. amygdalina* (n=10); the range of leaf width (cm) is 1.5–6.4 (4.2±0.6, n=17) vs. 3.5–5.2 (4.1±1.2, n=10). On the other hand, the two species are distinct in aspect ratio: 1.38–2.28 (1.79±0.25) in the Kaeng Krachan taxon vs. 2.46–3.43 (2.88±0.37) in *C. amygdalina*. The two taxa were also different in circularity, but with overlapping values: 0.55–0.77 (0.69±0.07) vs. 0.42–0.61 (0.51±0.06). Figure 4 shows that the Kaeng Krachan taxon is distinguishable from *C. amygdalina* by having a lower aspect ratio and larger circularity.

The abaxial leaf blade surface of the Kaeng Krachan taxon is sparsely covered with minute hairs that are almost invisible to the naked eye or hand lens (10 ×), but visible under a microscope (25 ×). Similarly, the lower leaf surface of *C. amygdalina* (T3090) is sparsely covered with minute hairs that are visible only under a microscope (25 ×). Both *C. amygdalina* and the Kaeng Krachan taxon have scalariform to scalariform-reticulate tertiary veins and it is difficult to distinguish between the two species by their venation.

The specimen T2069 of the Kaeng Krachan taxon had smaller fruits than the specimen T3090 of *C. amygdalina*: the range (and average±SD) of fruit length (mm) is 9.88–13.82 (11.49±1.28, n=14) vs. 22.07–28.1 (25.67±2.3, n=6), and the range (average±SD) of fruit width (mm) is 5.81–9.67 (7.53±2.36, n=14) vs. 11.27–12.63 (12.03±0.49, n=6). However, the fruits of T2069 (Fig. 2E) and T3090 (Fig. 3E) were green and not fully matured when collected. While the fruits of the Kaeng Krachan taxon are ovoid (Fig. 2E, F), the fruits of *C. amygdalina* are ellipsoid (Fig. 3E, F) but fruits of both specimens are still immature. Fruit stalks of *C. amygdalina* (T3090) are smooth and not or only slightly swollen (Fig. 3E, F) as in the lectotype (*Wallich Cat. 2585*, K001116509) and isolectotype (*Francis 990*, E00393147) of *C. amygdalina*. On the other hand, fruit stalks of the Kaeng Krachan taxon (T2069) are rough and swollen (Fig. 2E).

### Phylogenetic analysis

**Figure 5.**
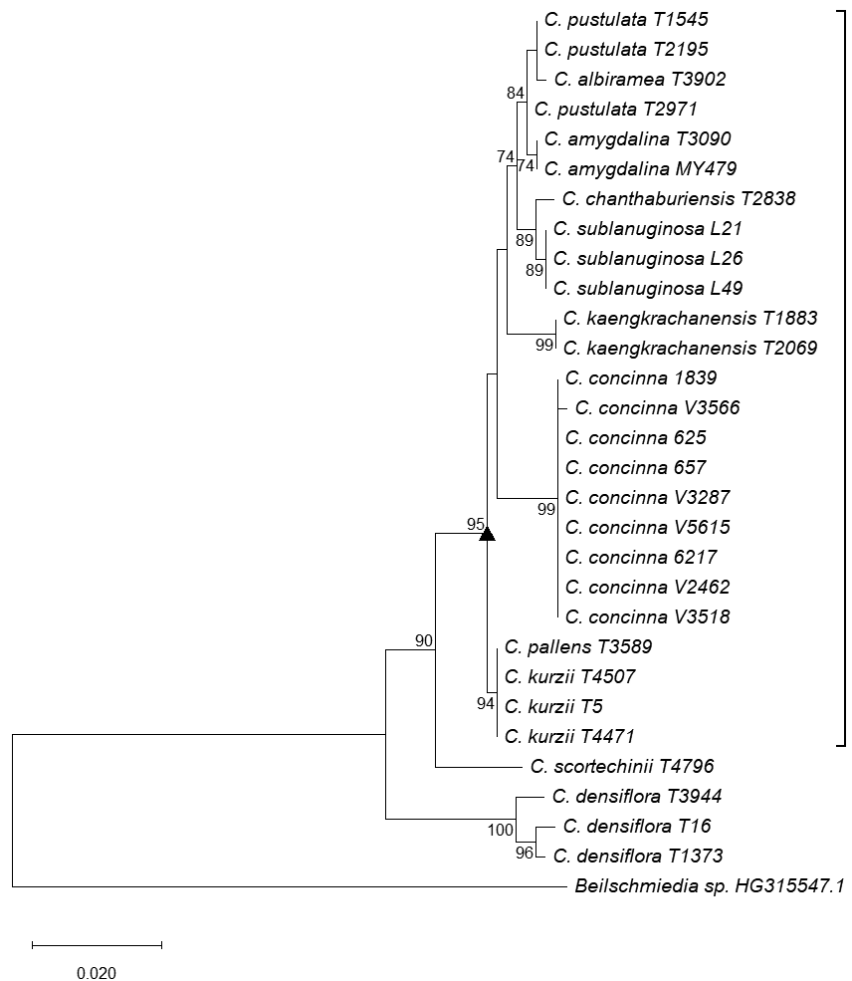
Maximum likelihood tree of *Cryptocarya* species from Thailand reconstructed from ITS sequences. Numbers: bootstrap values; Scale bar: mean number of nucleotide substitutions per site; solid triangle and square bracket: clade of 95% bootstrap value.

**Figure 6.**
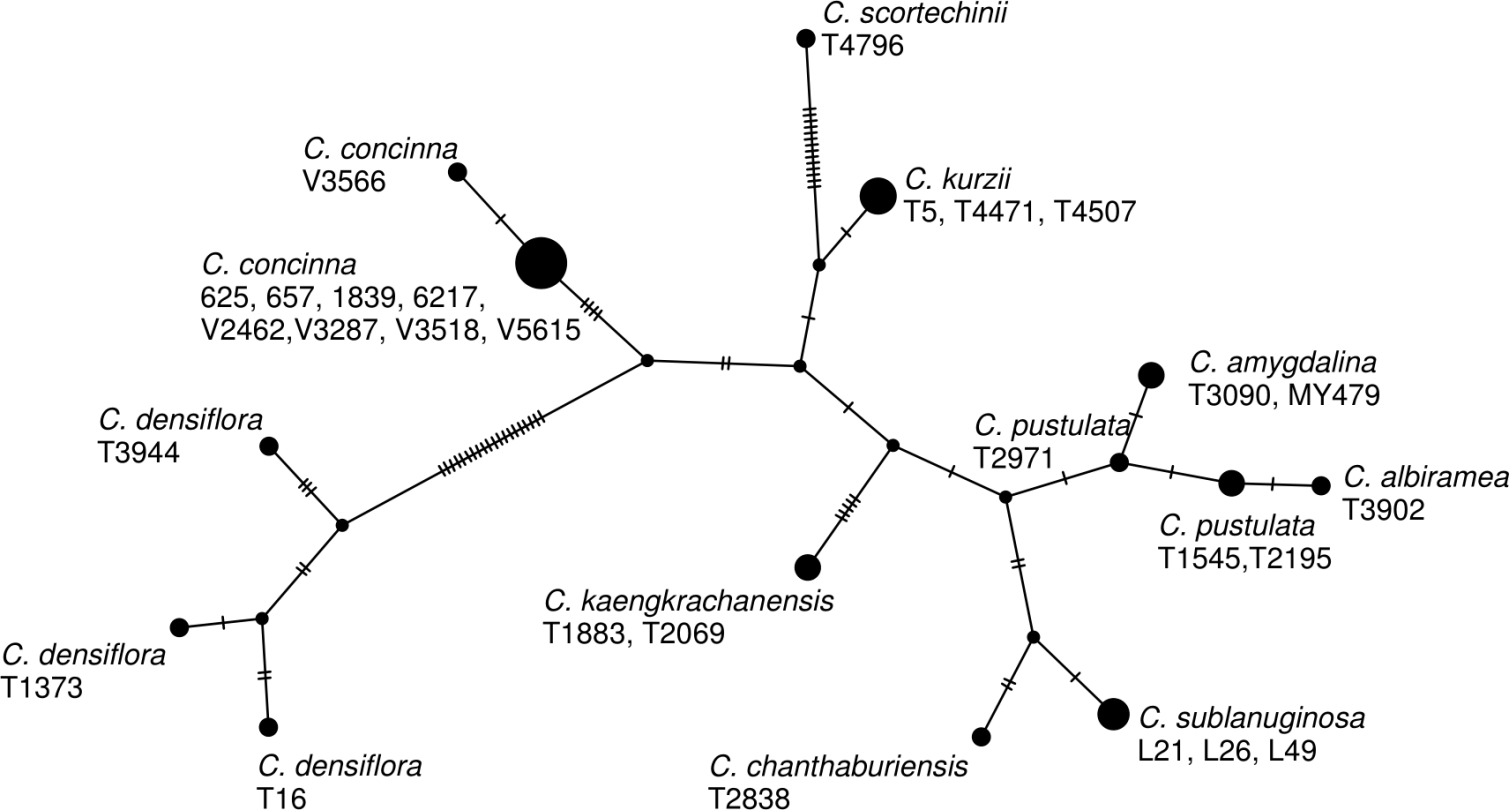
A haplotype network of *Cryptocarya* species from Thailand constructed from the ITS sequences.

A phylogenetic tree constructed from ITS sequences with length of about 670 bp (Fig. 5) showed that *C. amygdalina* is close to *C. albiramea* Kosterm. (T3902) and *C. pustulata*, and the bootstrap support for the monophyly of the clade including these species was 84%. For the ITS sequence, two samples initially identified as *C. amygdalina* (T3090 collected from Kaeng Krachan, Thailand and MY479 collected from Myanmar) were identical in the ITS sequences determined. Also, two samples of *C. pustulata* (T2195 of Kaeng Krachan and T1545 collected from Kao Soi Dao, Chanthaburi, Thailand, the type locality) were identical but another ITS sequence of *C. pustulata* (T2971) differed from T2195 in one base pair (Fig. 6). On the other hand, *C. amygdalina* is one species in a well supported clade with the plants of the Kaeng Krachan taxon sister to this clade, differing by 10 base pairs in the ITS sequences (Fig. 6) and are distinct from each other in the ITS haplotype network (Fig. 6).

**Figure 7.**
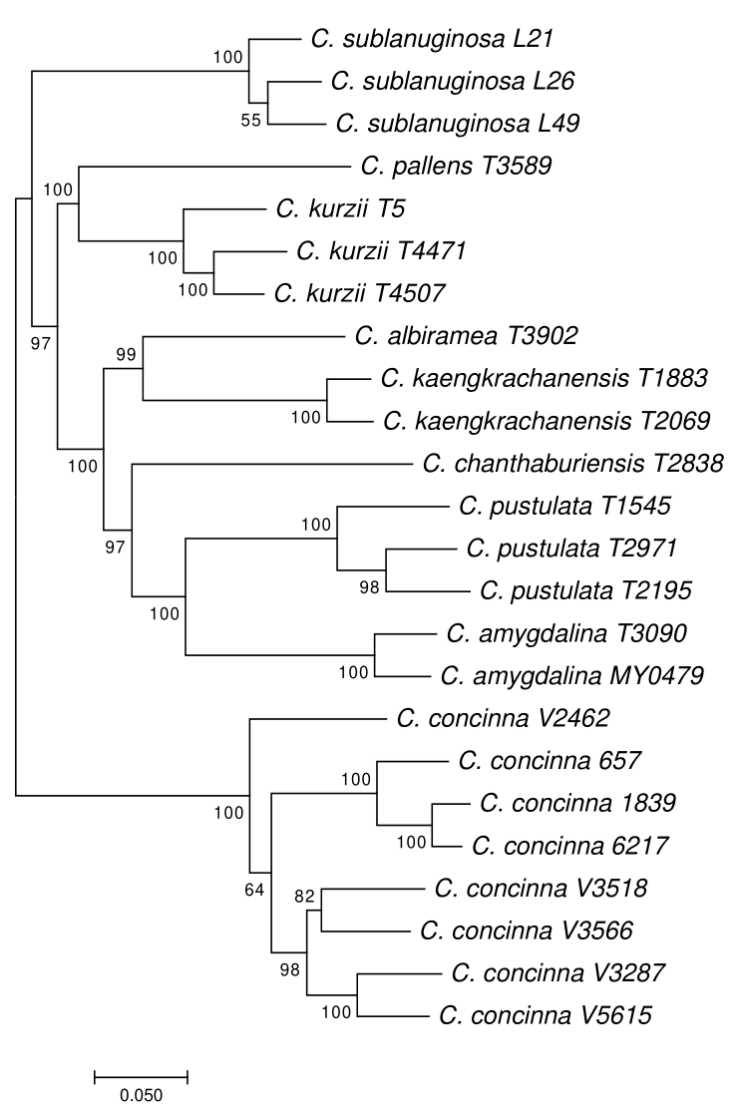
A maximum likelihood tree of *Cryptocarya* species from Thailand. reconstructed using MIG-seq data. Numbers: bootstrap values; Scale bar: mean number of nucleotide substitutions per site;

For 24 of the samples that belonged to a clade supported by 95% bootstrap value in the ITS tree (Fig. 5), we constructed a MIG-seq tree in which conspecific clusters of *C. amygdalina* (T3090, MY0479), the Kaeng Krachan taxon (T1883, T2069) and *C. pustulata* (T2195, T2971, T1545) were supported by 100% bootstrap values (Fig. 7). While *C. amygdalina* and *C. pustulata* were sister to each other, the Kaeng Krachan taxon was not sister to either of these species, but instead to *C. albiramea* (Fig. 7), with 99% bootstrap (BS).

Based on the evidence of morphology and phylogenetic analysis presented above, the two samples (T1883 and T2069) collected from Kaeng Krachan national park clearly represent a distinct and new species, which is named as *Cryptocarya kaengkrachanensis*.

### Taxonomy

#### Cryptocarya kaengkrachanensis

Taxon classificationPlantaeLauralesLauraceae

M.Z.Zhang, Yahara & Tagane sp. nov.

E8102981-1BD3-5137-800B-04A6D0B0B0C9

urn:lsid:ipni.org:names:77206953-1

Fig. 2

##### Diagnosis.

*Cryptocarya kaengkrachanensis* resembles *C. amygdalina* in having pinnately veined, leathery leaves apparently glabrous (microscopically hairy) below, young twigs with yellowish brown hairs and fruits 1.36–1.85 times longer than width. However, *C. kaengkrachanensis* differs from *C. amygdalina* (Fig. 3) in having the leaves ovate and elliptic to narrowly elliptic (vs. oblong-lanceolate) with leaf aspect ratio from 1.38 to 2.28 (vs. 2.46–3.43) (Fig. 4), and fruits ovoid (vs. ellipsoid) with the stalk distinctly swollen (vs. not or only slightly swollen). While *C. kaengkrachanensis* was sister to *C. albiramea* in MIG-seq tree, *C. kaengkrachanensis* is distinguished from *C. albiramea* by elliptic leaves with leaf aspect ratio less than 2.5 (vs. oblong-lanceolate leaves with leaf aspect ratio more than 2.5).

##### Type.

THAILAND. Phetchaburi Province: Kaeng Krachan National Park, 960 m elev., 12°49'19.7"N, 99°21'57.7"E, 23 Oct. 2013, *Tagane S.*, *Nagamasu H.*, *Naiki A.*, *Rueangruea S.*, *Suddee S.*, *Fuse K.*, *Keiwbang W.*, *Pansamrong P. T2069* [fr.] (holotype KYO!, isotypes BKF!, FU!, KAG!).

##### Description.

Trees up to 12 m tall. Young twigs densely covered with appressed short yellowish to brown hairs, old twigs lenticellate, terete and slightly hairy. Leaves alternate; blade leathery, ovate, elliptic to narrowly elliptic, the range (and average±SD) of leaf length (cm) is 2.6–10.3 long (7.2±2.5, n=17), the range (and average±SD) of leaf width (cm) is 1.5–6.4 (4.2±0.6, n=17) wide leaf aspect ratio from 1.38 to 2.28, obtuse or retuse at apex in adult trees, acuminate in young trees, broadly cuneate at base, green and not lustrous above and slightly glaucous below when fresh, brown above and grey brown below when dry, apparently glabrous but microscopically sparsely hairy below; pinnately veined, midrib sunken above, raised below, secondary veins 6 or 7 pairs, slightly sunken above, raised below, tertiary veins scalariform-reticulate, faintly visible above, raised below; the range (and average±SD) of petiole length (cm) is 0.7–1.5 long (1.1±0.22, n=10), flat above, rounded below, dark brown when dry, covered with short yellowish hairs. Inflorescences and flowers not seen. Infructescence axillary, 4–17 cm long (8.4±3.4, n=16) (the range, average±SD), rachis hairy, lenticellate; bracteoles not seen. Immature fruits ovoid, 9.9–13.8 mm long (11.49±1.28, n=14), 5.8–9.7 mm wide (7.53±2.36, n=14) with aspect ratio 1.36–1.85 (1.54±0.14, n=14). yellow green when fresh, dark brown when dry, shortly hairy. Fruiting stalk slightly swollen, rough, light brown when fresh, dark brown when dry. Mature fruits not seen.

##### Other specimens of *C. kaengkrachanensis* examined.

THAILAND. Phrae Province: between Ban Nam Krai and Pha Tuem, 16 Apr 1970, *Smitinand T.*, *Cheke A.S. 10817* [BKF 46511!]. Phetchaburi Province: Kaeng Krachan National Park, 960 m elev., 12°49'19.7"N, 99°21'57.7"E, 23 Oct. 2013, *Tagane S.*, *Nagamasu H.*, *Naiki A.*, *Rueangruea S.*, *Suddee S.*, *Fuse K.*, *Keiwbang W.*, *Pansamrong P. T1883* (BKF!, FU!). Kanchanaburi Province: Thong Pha Phum District, Pilok, at the Thai-Burmanese border. C. 900 m. 14 41.0'N, 98 21.8'E, tree 12m, 25 January 2009 [fr.], *Middleton D.J.*, *Karaket P.*, *Lindsay S.*, *Suddee S. 4785* [BKF 182421!].

##### Distribution.

Endemic to Thailand. The new species is currently only known in a few protected areas of Phrae, Phetchaburi and Kanchanaburi Provinces including Kaeng Krachan National Park.

##### Etymology.

The specific epithet *kaengkrachanensis* is derived from the name of the national park from which the species has first been recorded.

##### Conservation status.

Least Concern (IUCN 2012, 2017). This species occurs in hill evergreen forests of some protected areas including Kaeng Krachan National Park and there is no sign of declining trends.

## Discussion

In Kaeng Krachan National Park, we found three species of *Cryptocarya* that grew at different elevations. While *C. pustulata* was collected at lower elevations, 360 m, 550 m and 680 m, *C. amygdalina* and *C. kaengkrachanensis* were collected at higher elevations, 709 m and 960 m. *Cryptocarya pustulata* is a canopy tree constituent and attains a height of 25 m and we were unable to collect fertile material of this species. On the other hand, *C. kaengkrachanensis* is a subcanopy tree and we collected fruits from a tree 12 m tall. This species was common in the hill evergreen forest at an elevation of 960 m. *Cryptocarya amygdalina* and *C. kaengkrachanensis* are suspected to flower in different seasons because we collected a fruiting specimen of *C. amygdalina* (T3090) on 30 May 2014, and a fruiting specimen of *C. kaengkrachanensis* (T2069) on 23 Oct. 2013. The above observations in the field supported our hypothesis that there are three ecologically distinct species of *Cryptocarya* in Kaeng Krachan National Park.

In addition to ecological differences, the three species are genetically well differentiated. In particular, *C. amygdalina* and *C. kaengkrachanensis* differed by 10 base pairs of the ITS sequences and are placed in distant positions on both the ITS and MIG-seq trees. While *C. kaengkrachanensis* was sister to *C. albiramea* in MIG-seq tree, *C. kaengkrachanensis* is distinguished from *C. albiramea* by having elliptic leaves with leaf aspect ratio less than 2.5 (vs. oblong-lanceolate leaves with leaf aspect ratio more than 2.5).

To apply names to the species of *Cryptocarya* in Kaeng Krachan National Park, we examined the images of the lectotype and isolectotype of *C. amygdalina* and noticed that the description of the fruit morphology of *C. amygdalina* by de Kok (2015) does seem to not agree with the type material of *C. amygdalina*. While de Kok (2015) described the fruit stalk of *C. amygdalina* as “red, strongly swollen when mature” and used this state to distinguish *C. amygdalina* from morphologically similar species in the key, the type of *C. amygdalina* has fruit stalks not or only slightly swollen, as in our collection T3090. On the other hand, the fruit stalks of *C. kaengkrachanensis* are somewhat swollen, and brownish rather than red. The fruit stalks of *C. scortechinii* Gamble in Malay Peninsula are red and strongly swollen (e.g. G. Kedah, *T. Witmore FRI 4683*, KEP!). We suggest that the concept of *C. amygdalina* adopted by de Kok (2015) is a heterogeneous one that includes *C. amygdalina* s. str., *C. scortechinii* (see below) and *C. kaengkrachanensis*. In fact, the following specimen cited under *C. amygdalina* by de Kok (2015) is identical to *C. kaengkrachanensis* in leaf morphology; Phrae: between Ban Nam Krai and Pha Tuem, 16 Apr 1970, *Smitinand T, Cheke A.S. 10817* [BKF 46511]).

Before concluding that T2069 was an undescribed species, we needed to compare it with the type material of *C. floribunda* Nees and *C. scortechinii* Gamble, two names that were treated as synonyms of *C. amygdalina* by de Kok (2015). The type specimens of *C. floribunda* [Wallich Cat. n. 2593, BM000880687, K000768399, MEL2390468, MEL2390469, MEL2390467, MNHN-P-P02010447] have only flowers and we cannot verify the fruit characters. However, these specimens are most similar to the lectotype and isolectotype of *C. amygdalina* in floral and vegetative morphology. Thus, we agree with the earlier treatment of de Kok (2015, 2016a) that *C. floribunda* is a synonym of *C. amygdalina*. We collected a specimen (T4796) morphologically similar to the type specimens of *C. scortechinii* [King’s collector 6297, L0036248-lectotype, MEL2386583-isolectotype] at the elevation of 322 m at Khao Luang, peninsular Thailand. Although our collection is sterile, it is identified as *C. scortechinii* based on its leaf size, shape, and venation as well as its distribution in peninsular Thailand. As is shown in Fig. 5 and 6, *C. scortechinii* was placed in a distant position from *C. amygdalina*. Thus, our evidence does not support the treatment of de Kok (2015, 2016a) that *C. scortechinii* is a synonym of *C. amygdalina*. *Cryptocarya kaengkrachanensis* is easily distinguished from *C. scortechinii* by its elliptic leaves (aspect ratio lower than 2.5) that are obtuse at the apex and not lustrous above. Based on the evidence provided above, we here concluded that *C. kaengkrachanensis* is an undescribed species.

While de Kok (2015) included *C. amygdalina* in the group characterized by “Mature lower leaf surface glabrous, except on veins”, both *C. amygdalina* and *C. kaengkrachanensis* have minute hairs on the lower surface of leaves that are almost invisible to the naked eye and hand lens (10 ×), but clearly visible under magnification (25 ×). Because most species of *Cryptocarya* are more or less hairy on the lower blade surface and hairiness of leaves is very variable, we do not use the hairiness trait in the following key.

In his key, de Kok (2015) characterized *C. amygdalina* as “Tertiary veins scalariform” and other species as “Tertiary veins reticulate to scalariform”, but the specimen T3090 of *C. amygdalina* has undulate scalariform veins that are connected by finer reticulate veins. Among Thai species of *Cryptocarya*, *C. chanthaburiensis* Kosterm., *C. concinna* Hance and *C. densiflora* Blume are characterized by reticulate tertiary veins, but other species including *C. amygdalina* and *C. kaengkrachanensis* have more or less scalariform tertiary veins connected with finer reticulate veins. Thus, we used only two categories of venations, reticulate and scalariform, in the key that follows. In de Kok (2015), *C. diversifolia* Blume, *C. ferrea* Blume, *C. laotica* (Gagnep.) Kosterm., *C. nitens* (Blume) Koord. & Valeton, and *C. rugulosa* Hook.f. from Thailand, but these species are not included in the following key because we could not confirm the distribution of these species in Thailand.

### Identification Key to the species of *Cryptocarya* in Thailand

**Table:** 

1	Leaf aspect ratio less than 2.5	**2**
–	Leaf aspect ratio more than 2.5	**3**
2	Basal lateral veins attaining to 1/3 to 1/2 of leaf blade; tertiary veins reticulate; fruits globose	***C. densiflora***
–	Basal lateral veins attaining less than 1/4 of leaf blade; tertiary veins scalariform; fruits ovoid	***C. kaengkrachanensis***
3	Leaves (dried) distinctly glaucous below	**4**
–	Leaves (dried) not or only slightly glaucous below	**5**
4	Leaves lustrous above when fresh, distinctly foveolate above when dried	***C. albiramea***
–	Leaves not lustrous above when fresh, not foveolate above when dried	***C. kurzii***
5	Tertiary veins mostly reticulate	**6**
–	Tertiary veins scalariform	**7**
6	Leaves (thinly) leathery; lamina oblong, oblong-lanceolate, (5.5–)10–19 × (2.6–) 3–4.6 cm; petiole 0.8–1.5 cm long	***C. chanthaburiensis***
–	Leaves papery; lamina elliptic to elliptic-oblong, (3–)5–10(–13) × (1.5–)2–3(–6) cm; petiole 0.4–0.8(–1) cm long	***C. concinna***
7	Inflorescences longer than leaves; fruits ellipsoid	**8**
–	Inflorescences shorter than leaves; fruits globose or unknown (for *C. pustulata*)	**9**
8	Leaves lustrous above when fresh; fruit stalks thickly swollen	***C. scortechinii***
–	Leaves not lustrous above; fruit stalks not or slightly swollen	***C. amygdalina***
9	Leaves waxy below, light brown when dried	***C. pallens***
–	Leaves not waxy below, dark brown when dried	**10**
10	Finely reticulate veins raised on the upper surface of dried leaves	***C. pustulata***
–	Finely reticulate veins visible but not raised above	***C. sublanuginosa***

## Supplementary Material

XML Treatment for Cryptocarya kaengkrachanensis
